# A decade of aid coordination in post-conflict Burundi’s health sector

**DOI:** 10.1186/s12992-019-0464-z

**Published:** 2019-03-29

**Authors:** Johann Cailhol, Lucy Gilson, Uta Lehmann

**Affiliations:** 1 School of Public Health, University of the Western Cape, Cape Town, South Africa; 2 Laboratoire Educations et Pratiques de Santé, SMBH, University Paris 13, Bobigny, France; 3 School of Public Health and Family Medicine, University of Cape Town, Cape Town, South Africa; 4 Department global health and development, London school of hygiene and tropical medicine, London, UK

**Keywords:** Burundi, Post-conflict, Aid coordination, Power, Health sector, Donors, Global health initiatives

## Abstract

**Background:**

The launch of Global Health Initiatives in early 2000′ coincided with the end of the war in Burundi. The first large amount of funding the country received was ear-marked for human immunodeficiency virus (HIV) and immunization programs. Thereafter, when at global level aid effectiveness increasingly gained attention, coordination mechanisms started to be implemented at national level.

**Methods:**

This in-depth case study provides a description of stakeholders at national level, operating in the health sector from early 2000′ onwards, and an analysis of coordination mechanisms and stakeholders perception of these mechanisms. The study was qualitative in nature, with data consisting of interviews conducted at national level in 2009, combined with document analysis over a 10 year-period.

**Results:**

One main finding was that HIV epidemic awareness at global level shaped the very core of the governance in Burundi, with the establishment of two separate HIV and health sectors. This led to complex, nay impossible, inter-institutional relationships, hampering aid coordination.

The stakeholder analysis showed that the meanings given to ‘coordination’ differed from one stakeholder to another. Coordination was strongly related to a centralization of power into the Ministry of Health’s hands, and all stakeholders feared that they may experience a loss of power vis-à-vis others within the development field, in terms of access to resources.

All actors agreed that the lack of coordination was partly related to the lack of leadership and vision on the part of the Ministry of Health. That being said, the Ministry of Health itself also did not consider itself as a suitable coordinator.

**Conclusions:**

During the post-conflict period in Burundi, the Ministry of Health was unable to take a central role in coordination. It was caught between the increasing involvement of donors in the policy making process in a so-called fragile state, the mistrust towards it from internal and external stakeholders, and the global pressure on Paris Declaration implementation, and this fundamentally undermined coordination in the health sector.

**Electronic supplementary material:**

The online version of this article (10.1186/s12992-019-0464-z) contains supplementary material, which is available to authorized users.

## Introduction

The need for and complexity of aid coordination is well recognized in the literature as well as in policy debates [1–5]. Assessments of coordination since the late 1980s have also shown that the commitment of all actors to coordination principles and the principles’ adaptation to the context are essential to their implementation [6–8].

However, while the commitment to coordination exists the practice remains elusive for numerous reasons, among them stakeholders' different agendas and development visions [6–10] as well as limited capacity of recipients to coordinate [8]. Dodd et al., in a study of human resources for health and aid effectiveness agenda in Lao discuss this as an ‘intent-practice gap’ in donors’ and government’s engagement with the coordination agenda [11].

Among donor organizations there exists an inherent tension between accountability towards their home agencies (eg high income countries' governments or related agencies) and recipients’ priorities and agendas [12, 13]. Daniel Esser, for example, in a 2014 article on the tautological use of the ownership concept, argues (quoting [14]) that “donors, […] despite ‘proclaim [ing] in unison the importance of national ownership of the development process by recipient countries’ ([14], p. 180), ultimately put their own accountabilities first” ([15], pp. 48). One of his key arguments is that ownership is a tool created and used by donors to retain their power while shifting the responsibility for the lack of aid effectiveness to recipient’ governments.

For recipient countries, particularly in post-conflict setting, the literature has identified a number of missing ‘inner’ attributes required for coordination: ownership, but also institutional capacity, leadership, legitimacy, trustworthiness [13, 16–22]. In many cases it is donors, who have such negative perceptions of government capacity and integrity, and believe that government is unwilling or unable to coordinate, raising an inter-related issue of legitimacy and trustworthiness [22]. An evaluation of the applicability of the Paris Declaration on aid effectiveness to post-conflict countries particularly referred to the “weak institutional capacity” as a source of lack of coordination and therefore of insufficient aid effectiveness [23]. Furthermore, fragile and post-conflict states often lack local champions and leadership due to ‘brain drain’, decreased quality of training, high turnover, which all undermine good policy formulation and directly affects coordination capacity [24].

Moreover, the sudden influx of donors in the post-conflict period and their heterogeneity have been reported to render the coordination process difficult [8]. Severino and Ray have even suggested that “in some cases, the gains from having more actors involved are outstripped by the losses that stem from policy incoherence and coordination costs” ([25], pp.6].

Fragile and post-conflict states hence experience great challenges in aid coordination, especially with the growing number and diversity of partners in international aid, including the new Global Health Initiatives (GHIs). However, to date, little attention has been paid to the (internal and external) power relationships involved in the coordination process and to the extent to which these might influence coordination, while coordination necessarily implies power.

This paper presents an in-depth study of the aid landscape in Burundi following the end of the civil war, offering a different, and far less studied perspective on the discourses and practices of coordination. It considers what happens within a country and government when large sums of money begin to flow at the end of a civil war, and multiple donors jostle for influence. More specifically, it describes these dynamics by providing a historical and longitudinal analysis of aid coordination from the immediate post-conflict period, in early 2000, and the following decade.

Our analysis paints a complex picture of inter- and intra-governmental politics and power struggles, that impact on and are impacted by donor positions, leading to an active and passive undermining of national and global policy intent and practice, and subverting coordination from a functional point of view. This paper extends research and analysis of the role and impact of aid and aid coordination reaching back to the 1990s when policy researchers started discussing coordination as an essential instrument to improve aid effectiveness in the health sector [9, 26], and multiple mechanisms were devised and assessed [6–8] [9, 15, 27]. Buse emphasized the political and contested nature of aid and co-ordination in 1999, arguing that, “although lip service is paid to the over-used notion of ‘putting the recipient in the driver’s seat’ a conspiracy of interests, suspicion, development practices and inertia prevents this” (p 219). Seventeen years later Esser (2014: 50) showed that “recipient country preferences are [still] followed only to the extent to which they do not conflict with donors’ priorities”.

Attention on aid coordination somehow decreased during the global shift toward Millenium Development Goals and the launch of GHIs. It returned to the agenda with the Paris Declaration on aid effectiveness in 2005, and the increasing focus on health system strengthening. Indeed, it became clear that health system effectiveness was closely linked to aid coordination, as already pointed out by some authors in the 1990s [4, 22]. Since a lack of coordination was seen as related to the asymmetry of power between donors and recipients (thus exacerbated in fragile and post-conflict countries), the literature began to focus increasingly on an analysis of recipient country capacity (eg Brinkerhoff…..). A natural development of aid coordination studies after the launch of Paris Declaration was the analysis of the constituencies of the Paris Declaration: leadership and ownership of recipient countries [28–32]. All these studies show a rather superficial ownership from governments, and calls for an in-depth analysis of historical power (im)-balance at country level.

The paper seeks to extend these analyses and begins by painting the historical and institutional landscape of aid and aid coordination after 2001, before taking a closer look at roles and positions of internal and external organizations, as well as context and process-related factors influencing coordination. Its unique contribution lies in the fine-grained analysis of the immense complexity of relations between donors, between donors and government institutions, and, very importantly, within government itself, all of which further contributed to paralysis, fracturedness, mistrust and inefficiency in an immensely fragile post-conflict country.

## Methods

The design of the study was qualitative in nature, retrospective, and used two different sources of information: semi-structured interviews with national and international role players (details of the interview schedule is provided in a Additional file 1) and documents analysis.

Nineteen organizations, active in 2001, the year of approval of the first funding from GHIs, were selected according to 2 criteria: exerting a direct or indirect influence on health policies at any step of the health policy cycle and at national level; and providing a financial contribution to the health system.

The following organizations were included in the study, each organization constituting one unit of analysis, as well as the whole set of organizations: the Ministry of Health (MoH) central (named “central” by contrast to vertical programs); vertical programs/unit within MoH (malaria, tuberculosis, reproductive health, immunization programs and HIV unit) as separate stakeholders from MoH central; bilateral and multilateral donors (UK Department of International Development – DfID-, Belgian technical cooperation –BTC-, European Union –EU, World Bank); GHIs such as Global Fund for AIDS Tuberculosis and Malaria (GFATM), Global Alliance for Vaccine and Immunizations (GAVI), GAVI-Health System Strengthening (GAVI-HSS); Permanent Secretariat of National AIDS Council (PES-NAC); Ministry of AIDS (MoA); United Nations for AIDS (UNAIDS) and World Health Organization (WHO).

Interview participants were selected based on the relevance of the position they held in their organization to the topic of the study and/or further identified through sequential reference/snowball sampling.

Data collection was conducted between February and June 2009, as part of the WHO study “Maximizing positive synergies between GHIs and health systems”. The main researcher collected data together with two research assistants recruited locally and trained for the purpose of the study.

Overall, 29 people were interviewed (Table 1). A stakeholder perception and position analysis was conducted in order to assess actor-related factors inhibiting coordination. A framework analysis was used, according to the following themes: knowledge of, interest in /position towards and perceived barriers to coordination. This analysis was part of a wider analysis of aid coordination in the health sector in Burundi, including process and context analysis [33].

**Table 1:** List of interview participants, by organisation

Organization	Number of local participants	Number of expatriate participants
MoH central	4	2
MoH vertical programs	5	
BTC		1
DfID		1
European Union	1	1
World Bank		1
MoA	3	
PES-NAC	3	
UNAIDS	1	
WHO	1	1
GAVI-HSS	1	
Other (local NGO)	1	
GFATM	2	
Total	22	7

The language used for data collection was French. All transcripts were translated in English, to enable cross-comparison of themes with other researchers of the team at the University of the Western Cape, School of Public Health. Themes were presented to the 2 research assistants in Burundi, to validate interpretation consistency.

Quotes throughout the paper are identified by category of participant and its origin (local or expatriate). The origin of participant was important to distinguish, since expatriates were more open in their viewpoints and less prone to implicit meanings.

Material for document analysis were collected during the interview process, or downloaded from partner’s institutions websites. They were also analysed using the same framework analysis, and provided details on the organizational history. They are listed in Table 2.

**Table 2:** List of documents consulted, according to the organization

Organization in charge of document elaboration	Policy and planning documents consulted	Corresponding evaluation documents consulted(independent or not)
MoH central	National Health Plan 2005–2015 National Health and Development Plan 2006–2010 and its attached financial plan HRH pay-for-performance policy (2006)	Report on technical assistance for HRH management (2005) HRH analysis from MoH-WHO (2008) Evaluation of national health plan 2006–2010 (2011)
National TB and Leprosy Program	National TB strategic plan 2007–2011	Annual activities reports on TB program 2007–2008
Health sector HIV unit	National HIV unit plan 2008	National HIV unit activities report 2007
National Malaria Program §	National Plan against Malaria 2008–2012	NA
National Reproductive Health Program	National Reproductive Health Strategic Plan 2008–2012	Annual reports on national reproductive health program 2005 to 2007 (no plan)
National Immunization Program / IACC (GAVI)	GAVI /Immunization Program Multi-annual Plan 2002–2006 GAVI /Immunization Program Multi-annual Plan 2007–2010 GAVI / Immunization Program Annual Plan 2007	Immunization plan /GAVI annual report 2006
BTC	Indicative collaborative program 2003–2005 Technical and financial document for Kirundo project Institutional support project document	Analysis of BTC contribution to Burundi 1996–2002 (OAG)
DfID	NA	NA
European Union	EU Cooperation Strategy and National Indicative Program 2003–2007 LRRD ‘santé plus’ project document (EDF10)	EU-Burundi Joint annual report (2002) Note on the situation in Burundi-UE (2007)
World Bank	Second Health and population project (PSP2)* MAP1 proposal	PSP2 report* Final report on MAP1 implementation MAP1 implementation completion and results report* (2009)
MoA	No specific document available	NA
PES-NAC	National Strategic Plan against HIV 2002–2006* National Strategic Plan against HIV 2007–2011 National Strategic Plan for HIV M&E 2007–2011	Annual activities reports of national strategic plan against HIV: 2003 to 2008 Mid-term review of the implementation of the national strategic plan against HIV 2002–2006 (2005)
GFATM/CCM	Funding proposals for: GFATM-HIV (RIBUP, 2003–2006* and APRODIS, 2006–2010) GFATM-malaria 2003–2006 and its RCC 2006–2008 GFATM-TB 2005–2010	Grant performances reports GFATM-HIV* GFATM-malaria* GFATM-TB* CCM retreat report 2008 Annual report on the RCC 2007–2008 (GFATM-malaria)
WHO	WHO Country Cooperation Strategy 2005–2009 WHO Health Sector Reform Strategy (2006)	NA
GAVI-HSS	Proposal for GAVI-HSS support (2006)	GAVI-HSS evaluation (2009)
UNAIDS	3 by 5 Initiative 2004–2005	NA

*All documents were written in French except for those marked, which were written in English

## Findings

### Historical context

The post-independence history of Burundi, after 1962, has comprised 40 years of overt or latent armed and political conflicts between different social groups, which have their roots in the country’s colonial history. The last civil war was triggered in 1993 by the assassination of the first democratically elected Hutu President. In 2005, after a long peace negotiation process driven by external actors in Arusha [34], a constitutional democracy was established. Article 129 of the constitution importantly legislated proportional representation of the 2 main ethnic groups in all governmental positions (60% Hutus and 40% Tutsis), in order to facilitate inter-ethnic reconciliation [35]. This reconciliation process is quite specific to Burundi: externally driven peace negotiations are quite frequent and impose a certain type of governance; but engraving ethnic quota is unique and must be interpreted in the Great Lakes regional history of continuous power disputes and mass violence. While this was an important step in an effort to heal the deep rifts in Burundese society, it did not expunge the deep distrust permeating the country and all its institutions. It also immensely complicated the rebuilding of institutions, as human resources capacity was extremely limited in the aftermath of the war, and now had to be proportionally constituted. In 2010 and 2015, presidential election results were contested [36], and accusations of corruption grew louder [37]. In 2018, the constitution was changed, by removing some ethnic requirements such as setting 2 vice-presidents from different ethnicities.

The influx of aid, and the proliferation of actors and interests in health have to be seen against this fragile national context, including the competition for resources. The sudden inflow of post-conflict aid coincided with the global launch of GHIs and the United Nations General Assembly Special Session (UNGASS) declaration on HIV, creating an extraordinary context for aid in the health sector in Burundi.

### A sign of its time: The HIV silo

As peace negotiations began in early 2000′, donors began to position themselves and pour into the country. This timing coincided with the UNGASS declaration in 2001 and related international pressure, which gave high political visibility to HIV. In response to these global requirements, and influenced by the World Bank, whose discourse was to consider HIV not as a solely health issue but as a wider development issue, the government of Burundi, and its major donors established HIV as a stand-alone sector, separate from the health sector, in the poverty reduction strategic paper. The scope of HIV was considered to go beyond the scope of the MoH, whose mission was to deal “only” with curative health issues, via its « HIV unit ».

* “….. The World Bank also realized that the HIV issue was wider than simply a medical problem, but was rather a multisectoral and behavioral issue, and that such an issue could not be dealt by the MoH alone. The MoH was at that time seen as very technical and in particular not necessarily able to disseminate prevention messages.” (donor representative 1, expatriate).*

Two major institutional reshufflings occurred subsequently. The first was the creation of a separate MoA in 2002, directly linked to the Presidency, encouraged by local WHO and UNAIDS. This was meant to assure donors that Burundi was taking into account the need to tackle HIV in a cross-sectoral way. Secondly, the National AIDS Council (NAC) and its Permanent Secretariat (PES-NAC) were launched in 2002, after a joint decision of the World Bank and UNAIDS, on the need of a unique body of coordination for HIV. The Country Coordination Mechanism (CCM) was soon nominated. These were the first steps to enable application for HIV-related funding, such as those proposed at global level by the World Bank (Multisectoral AIDS Project 1 - MAP1) and the GFATM. In fact, initial non-emergency funding in the health sector in Burundi was mainly represented by GHIs, such as GFATM, the MAP1 of the World Bank and GAVI.

As a result four main national-level institutions dealt with HIV activities from 2003 onward: CCM, MoA, HIV unit of the MoH and PES-NAC. These newly created institutions were tied together by complex relationships and changing functions, sometimes competing against each other thus inhibiting communication and coordination. PES-NAC established very tight financial management mechanisms since GFATM and MAP1 disbursements were conditional on the approval of regular reports from the recipients. Though MoH remained theoretically in charge of implementing curative HIV activities, de facto PES-NAC progressively became an implementing agency.

And when donors imposed some requirements upon PES-NAC contracts, these were dismissed.

*“Unfortunately, the government recruited people on salaries we did not want. (…) The PES-NAC recruited people for M&E and we required them to sign a contract with the government (…). But it was difficult due to issues specific [referring to ethnic quota] to Burundi”* (donor representative 1, expatriate).

This parallel financial management further contributed to the MoH being sidelined, reflecting at least doubts about the capability of the MoH to deal with such complex and multisectoral issue as HIV. As a result, the MoH resented the political importance given to the MoA and the financial importance given to PES-NAC: the MoH had no legitimate role to manage Non-Governmental Organizations (NGOs), while PES-NAC relied on NGOs to implement HIV activities. Besides, the MoA would say “Health is not my issue, mine is development” (UN representative, expatriate). A further complicating factor was the fact that during the conflict, the landscape of health sector funding was dominated by emergency and humanitarian aid. Aid for development, including for the health sector, became available progressively after 2001, when major battles calmed down. Service delivery during the war was dominated by NGOs. In the early 2000s many of these switched their focus to HIV, and proliferated. By 2008 over 500 NGOs were active in the country in HIV-related activities [38, 39]. These were primarily accountable to their donors and almost impossible to control or coordinate by government.

### The donor landscape

Beside GHIs, a number of donors operated in Burundi, some since its independence, and all with different financing mechanisms, operating procedures, interests, aims, objectives, visions and historical alliances. Amongst major donors, the Belgian Technical Cooperation (BTC) was the largest bilateral and the oldest. It mainly operated via projects and technical assistance. British DfID, having arrived in 2004, was the newest, and supported coordination in the health sector via technical assistance. The World Bank and the EU had been active in Burundi since its independence in 1962. The World Bank operated via projects, and historically contributed to both HIV (with its MAP 1 and 2 funding after 2001) and health sectors (before 1993 –health population projects). The EU also operated via projects and technical assistance. The jostling for political influence through aid was very tangible when analyzing the operational procedure of funding agencies. There were several examples in our findings to illustrate each actor’s particular interests in securing Burundi as a privileged bilateral partner. For example, during the conflict, the EU and the World Bank continued to operate, presumably in order to secure a return on investment in the post-conflict period. Historical linkage and geo-political interests also influenced eligibility to aid, such as for BTC, following a high-level decision to focus development aid in Central African countries.

BTC and DfID, and to a much lesser extent WHO, were the only donors directly supporting the MoH central. WHO or UNAIDS funded vertical programs occasionally, to cover financial gaps or in the case of emergencies (such as epidemics) and WHO provided technical assistance for policy development.

GFATM in Burundi cannot be considered a single organization but rather as three different organizations, corresponding to each of the three diseases to which it contributed. While the three components had similar principles, especially with regard to funding application procedures and eligibility, management differed greatly from one component to another. The difference was significant with respect to the selection of the principal recipient (PR) by the GFATM: while the National TB Program became logically the PR for the GFATM-TB grant, the PES-NAC was given the role of PR for GFATM-HIV grant (together with the MAP1 and 2 grants) and, more surprisingly, also for GFATM-Malaria.

**Fig. 1:**
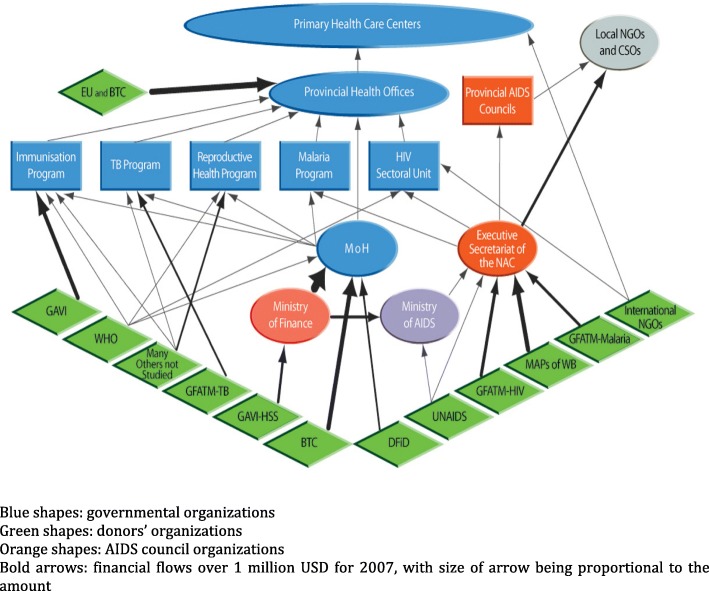
Synthesis of financial channels between the selected 19 organizations and some other key organizations (local NGOs, Ministry of Finance), as of 2008 *(own creation).* Blue shapes: governmental organizations. Green shapes: donors’ organizations. Orange shapes: AIDS council organizations. Bold arrows: financial flows over 1 million USD for 2007, with size of arrow being proportional to the amount.

The complexity of institutional arrangements in government, together with a diverse donor landscape, generated an enormous web of financial channels and relationships, which is presented in Fig. 1.

**Fig. 2:**
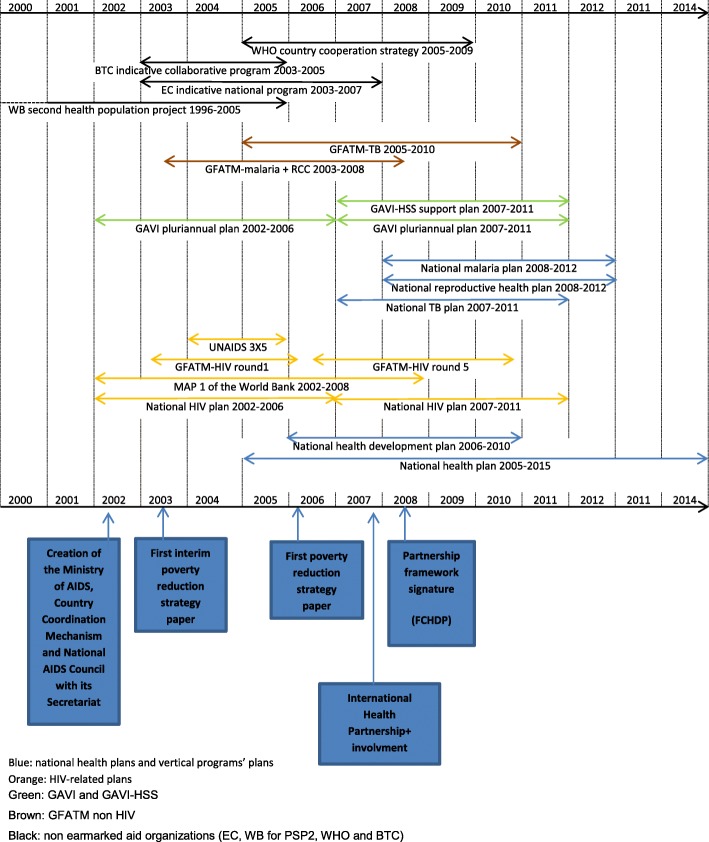
Timeline of major institutional reshufflings, policy and planning documents and grants in the health sector between 2001 and 2010 (own creation). Blue: national health plans and vertical programs’ plans. Orange: HIV-related plans. Green: GAVI and GAVI-HSS. Brown: GFATM non HIV. Black: non earmarked aid organizations (EC, WB for PSP2, WHO and BTC)

All these organizations had their own planning processes, following grant applications periods, and the first post-conflict plans for vertical programs started before the national health plan was launched in 2005 (Fig. 2).

Under the influence of the Paris Declaration and the Accra Agenda for Action, two coordination mechanisms started to operate in Burundi, in addition to the pre-existing CCM and MoH directorate of programs: the National Committee for Aid Coordination (NCAC), created in 2005 by the President of Burundi, under the influence of major donors; and the International Health Partnership Initiative (IHP+), from 2007, pushed by local WHO.

### National and international coordination mechanisms

#### A national level coordination mechanism: The framework for consultation of health development partners

The NCAC supervised 13 sector groups, of which one was for HIV and another for health, reproducing the distinction between HIV and health sectors created by the poverty reduction strategic paper. In the health sector, one major achievement was the establishment of a framework for consultation of health development partners (FCHDP) in March 2007, pushed by the Minister of Health at that time and DfID.

Following the creation of this framework, a Memorandum of Understanding was signed in February 2008 [40]. The first signatories were EU, the World Bank, DfID, Swiss cooperation, 4 NGOs, some UN agencies and the MoH. Expected intermediate outcomes of this Memorandum included an « improved efficiency of international aid » via the commitment of financial and technical partners and the MoH to “increased coordination and consultation” [40]. This framework also introduced the creation of a permanent technical multi-sectoral organ, in charge of piloting and monitoring progress using Millennium Development Goals indicators. Monthly meetings of the technical FCHDP were co-headed by the Minister of Health and the lead donor in the health sector. The first meeting of the technical FCHDP was held in February 2009, but the regularity of meetings seemed to depend on other external factors. Some topics, suddenly prioritized, took over time and resources available. For instance, between May and December 2009, no meeting was held, since Pay for Performance (P4P) became the policy priority, led by a managing unit created *ad hoc* at MoH central level ([41] and donor representative 2, expatriate). Moreover, FCHDP meetings were open to more than 50 individuals, with the aim to be as inclusive as possible. While the heterogeneity of actors could be an added value, the absence of hierarchy and rules in terms of decision-making did not favor coordination. Analysis of meeting proceedings made the meeting look more like an information sharing platform.

#### IHP+ initiative: A global response to Paris declaration in the health sector

The IHP+ (renamed UHC2030 since 2016) was a global initiative launched in 2007 in order to catalyse coordination in the health sector and help achieve the MDGs. The first step was a signature of a global compact, which included main bilateral donors (but not USA) and GHIs such as the World Bank and GFATM. The aim of IHP+ was to lead to the signature of a ‘compact’ between government and all development partners in the health sector at national level [42]. Practical actions in order to better coordinate activities in the health sector, linked to the IHP+, included, in Burundi: an attempt to harmonise P4P initiatives which were unofficially and officially on-going all around the country; the elaboration of the first national health expenditures estimates for 2007 (finalised in 2009); and the decision to mainstream HIV-earmarked funding (part of MAP2) from the PES-NAC to the MoH central.

According to one IHP+ progress review conducted in 2009, sector dialogue remained very weak, however, essentially conducted by donors. Disbursement of IHP+ funds to Burundi was very low (8% of US$800,000 over the first two years) due to the heavy demands of administrative procedures and the lack of ownership by government [41]. The lack of skills and capacity to coordinate was acknowledged both by the donors and government [41, 43]. Government did not grasp the purpose of IHP+ initially and managed IHP+ « catalytic funding » as another « project ». Joint Monitoring &Evaluation (M&E) missions of key donors started to be organized annually from 2007, with uneven participation. The DfID, the EU and BTC were the lead donors in joint M&E missions, but were not necessarily followed by others, especially the GHIs, despite the fact that they had signed the compact at global level. Eventually, DfID withdrew its aid from Burundi in 2012. In contrast to other GHIs, the World Bank shifted its attitude and got involved in joint missions from 2011. The World Bank's new policy was to not renew the MAP funding (which meant cutting the budget for recurrent cost of PES-NAC) and to focus on health system financing, through the P4P mechanism. The FCHDP was supposed to act as a vehicle for conveying coordination using IHP+ as an overarching process, but instead, FCHDP used IHP+ and its tools as another outcome-based project.

#### Proliferation of coordination mechanisms without coordination

Beside IHP+ initiative and the FCHDP, other coordination mechanisms existed. Since its creation in 2000, the MoH, the MoA, the MoH HIV unit and PES-NAC members participated in CCM, making it the only formal place where all of them eventually sat together regularly to discuss HIV. On paper, one of the CCM’s roles was to ensure linkages and consistency between GFATM assistance and other development and health assistance programs in support of national priorities, such as poverty reduction strategies or sector-wide approaches [44]. However, in Burundi, CCM’s role (to which everyone adhered) was limited to proposal development, around the three diseases targeted by the GFATM. The CCM could have been used as a space for coordinating HIV and health sectors. It could also have acted as a steward for mainstreaming part of HIV activities into the health sector. Instead it remained a simple fundraising instrument. By contrast, Mozambique for instance managed to include GFATM and therefore the CCM in a sector-wide mechanism, despite all the heavy reporting requirements attached to GFATM and despite also being a post-conflict country [45]. In Burundi, CCM was the first so-called coordination mechanism in place and given the two-headed nature of health and HIV sectors, it proved unworkable. Other coordination mechanisms were created around 2005, such as extended UNAIDS working group and its coordinating group, but had short survival periods.

There was also an attempt to merge MoH and MoA in November 2007, and the MoA became a vice-Ministry of AIDS within MoH. The MoA reversed back to a stand-alone Ministry in January 2009, and was definitively merged with MoH in July 2010.

Eventually, the Directorate for Programs and Health Services (from MoH central) was, in theory and on paper, in charge of coordinating vertical programs, including HIV unit, malaria and tuberculosis programs. In practice, the only role the Directorate played was to ensure that two programs did not clash physically during their in-service training provision. Since vertical programs managed their own funding, or asked for disbursement to PES-NAC (or to the Minister Cabinet, which managed some funding directly), they by-passed the Programs Directorate.

### Stakeholder perceptions and practices of coordination

#### Coordination: A desirable goal with differing scopes

While all stakeholders interviewed retained the discourse of co-ordination, perceptions and practices differed sharply. All interviewees from MoH, but vertical programs, identified the FCHDP as one, if not the main, tool for coordination.

Vertical programs did not seem to be convinced about the usefulness of the new FCHDP initiative. Interviewees perceived their participation in the FCHDP as unimportant, since it was not attached to any incentive and coordination was considered an inconvenience, costing time and energy. Also, they felt that non-participation in FCHDP did not have any consequence for program functioning, since programs were self-governed (by legal statute or by custom).

The majority of respondents, except those from GHIs-related organizations, argued that it was impossible to coordinate with the MoA and PES-NAC since the HIV budget was separate from the general health budget.

*“This funding (MAP1) was politically oriented. It had a multisectoral dimension and gave a very limited role to MoH. (…). Throughout almost all of MAP1, the MoH was particularly absent. The MoA held the key for funding (…). As much as USD36 million was made available […]* (NGO representative, local)”.

Vertical program managers understood coordination as centralization of financial resources into the coordinator’s hands and hence perceived coordination as a loss of power, while local actors considered having a hand in the financial management a *sine qua non* condition for being able to coordinate. Vertical programs, which used to self-manage their funding, were accountable to donors regarding the use of funding. While they were willing to coordinate, this was only within their program’s scope, so as to keep a tight grip on funding.

*“Program directors do not want to be integrated nor coordinated; they want to keep their independence in terms of management” (UN representative, expatriate).*

In fact, no interviewees from vertical programs, structurally part of the MoH, declared seeing any benefit in being coordinated by one organ. Instead they expressed the feeling that they should ‘self-coordinate’ as a stand-alone program. No one was prepared to give up on their own resources, let alone into the hands of a general coordinator, in a country so poorly resourced.

A striking feature, however, was that even though vertical programs did not want to be coordinated, they criticized other organizations’ apparent opposition to coordination.

Overall, organizations blamed others for the lack of coordination, especially competing institutions - for instance the EU blamed non-EU member states.

The WB, one of the biggest funders since the independence of Burundi, was perceived as the strongest opponent to coordination by other actors and behaved as such when MAP1 funding was implemented, by operating in a silo. While the World Bank changed its policy and progressively shifted its focus to mainstreaming their MAP2 funding into the P4P implementation, it competed with the EU to impose its neoliberal ideology (policy advisor, MoH, local). Other donors still continued to accuse the World Bank of performing bilateral negotiations with the MoH and acting against coordination, despite being part of the FCHDP. The MoH actors defended however the World Bank clientelistic behavior against other donors:

*“I think that it has been a misunderstanding. […] It is true that the World Bank is proceeding more quickly than others today. And I think to some extent that it is also an opportunity to set up the tools and structures which make it possible for all the donors to sit together.” (policy maker 1, MoH, local).*

The way some donors negotiate bilaterally with government, together with the “laissez-faire” of uncoordinated management, might lie somewhere between a lack of capacity to coordinate and passive clientelism. The line is sometimes thin between these two notions, and the distinction would relate to what values drive MoH and its people, which are fundamental to understand [46, 47].

Only two interviewees mentioned IHP+ initiative as a coordination tool, and the signature of the compact at national level understood as a goal (and not as a process of coordination).

In addition to coordination challenges at inter-organizational level, donors and international observers considered the risk of a relapse of conflict in Burundi as extremely high. Donors were therefore extremely cautious and were not prepared to finance long-term plans. However short-term interventions made co-ordination all the more difficult, further undermining the co-ordination endeavor.

#### Mistrust towards and within MoH

Stakeholders in general, whether local or international, internal or external to MoH, agreed on one point: they did not trust the MoH capacity to lead or coordinate aid. There were lots of references to the “lack of leadership” of the MoH. Interviewees felt that for instance, despite their willingness, the Minister (or alternatively the director general for Health who represented the Minister), did not have any power to insist that parties participate in FCHDP meetings. Neither vertical programs nor GHIs representatives participated and none of them saw an advantage or incentive to be coordinated.

*“It is up to the Minister [of Health] to lead everyone and to call for the participation (…) to the FCHDP, since he is the head of all health programs. It is not the case today and all the [EU] partners and the EU do not understand why”.* (donor representative 3, expatriate).

There was clearly mistrust with regard to funding management, with the risk of mismanagement if funding was to be pooled and centralized mentioned specifically. Parallel financial management systems were set in place (such as PES-NAC), justified with a necessary swiftness of reporting, but *de facto* in order to prevent any grant mismanagement.

*“I am not of the opinion to adopt a ‘common basket’ approach. (…) Just put the money with people you can control! You cannot easily punish such important personalities as ministers, but you can put any program director into jail. The idea of using a ‘common basket’ at the level of the general directorate for resources is unreliable.”* (vertical program manager, MoH, local).

Interviewees from MoH central and others felt the Programs Directorate did not have any financial weight and that this was the justification for being bypassed and being unable to coordinate vertical programs.

MoH was not considered skilled enough also for technical reasons, all interrelated, amongst them the brain drain during the civil war, lack of knowledge of the vocabulary specific to funding, and time constraints attached to funding application or policy reform.

Therefore, there was an ‘externalization’ of the policy and funding proposal development processes, as illustrated by a generalized use of international consultants for the elaboration of national plans and grants applications.

*“Are we really aligned with national priorities? We need first to look at how these national priorities were elaborated, because in most weak capacity countries, an international expert arrives with a document that he copy-pastes and adapts a little. […] I discussed with some experts from the WHO, who wrote certain planning documents. They did everything from A to Z. When there is no capacity, someone needs to do the job.”* (donor representative 3, expatriate).

## Discussion

Even though formal commitment was present, Burundi internally faced multiple constraints inhibiting coordination, constituted by, inter alia: funding arrangements and financial accountability channels imposed by donors, ethnic quota, institutional reshuffling set at global level and attached administrative burden. Coordination mechanisms stood in direct competition with each other, such as in the case of FCHDP and CCM, making them “competitive and duplicative” [9] (Buse 1999: 224). Where expedient, they were simply ignored, most notably with the FCHDP, the reasons being, inter alia, a high transaction cost of coordination, and the complexities of processes. Most importantly, power imbalance between stakeholders both internally and externally, due to historical, political and financial context, fostered presumably clientelistic relationships, which subverted coordination.

### Accountability requirements in a post conflict period: Conducive to giving birth to “monsters”

Burundi emerged from years of conflict at the same time as the world’s aid focus shifted to HIV/AIDS. International institutions, governments and NGOs aligned their health agendas to gain access to the immense resources available for HIV/AIDS programming, framing what was funded, and how it was funded. The UN agencies’ “Three Ones” principles, agreed by donors and recipients of HIV-related funding shaped the very structure of government and governance of the health sector in many countries [48, 49]. Burundi was no different, starting by the distinction of HIV and health sectors in the poverty reduction strategic paper (another donor-driven policy at macro-level). In the health and development sector NGOs proliferated and, most crucially, a whole parallel system was set up to deal with HIV. The new agendas served as a basis for the creation of MoA and PES-NAC, and their attached parallel implementation and M&E structures. Convergence between global policies on AIDS exceptionality and the post-conflict context in Burundi contributed to create structural factors, which contributed to transform these organizations into implementers and undermined coordination. As one interviewee said when talking about PES-NAC: ‘we created a monster’ (donor representative 1, expatriate). In theory, GFATM funding constituted an innovation at its conception: the recipient was meant to manage the grant. However, just like in other countries [50], the way this funding was implemented in Burundi proved to be the opposite.

In his paper on capacity development in fragile states, Brinkerhoff emphasizes some aspects donors need to take into account: the length of financing, which has to be as long as possible; a pooled funding managed jointly by donors and government; and a minimal burden of grant management to prevent donors to by-pass governments [51]. Our analysis showed that none of these requirements were respected in Burundi during the last decade, especially when negotiating the poverty reduction paper and with regard to GHIs funding. Other authors proposed new tools specific to post-conflict countries, different from the traditional log-frames, which are not adapted [52]. Instead, they argue for integrated M&E tools which would be sensitive to culture, people and relationships [52], that would avoid perpetuating the same mistakes of implementing parallel structures and could also act on complex conflict roots.

### The vicious cycle of pervading mistrust, low capacity and struggle for power

Those actors who had the greatest power were the strongest opponents of coordination, since they perceived coordination as having a detrimental impact on their mandate and power. Lack of local capacity and trust, particularly in the MoH, became a key argument. Indeed, all actors who had a minimum of financial autonomy (e.g. the Reproductive Health Program, the TB Program and PES-NAC), argued that coordination would imply a centralization of resources in the MoH hands, with a subsequent decrease in their ability to mobilize funding. By-passing the MoH was a normal process during the war, and was perpetuated in its aftermath. This distrust towards the state further weakened local governance, as noted by Uvin [46].

Another feature of the post-conflict period was the increasing involvement of donors in the policy making process. Indeed, donors not only participated in, but dominated policy processes from A to Z – from issue identification (in the national health forum) and priority setting (consultations between embassies, cooperation heads and government), to policy elaboration (grant and action plans elaboration support) and implementation (evaluation and monitoring by international NGOs, by joint donor missions and other donor-driven initiatives). Donors often took the lead in activities (as implementers or as policy makers, or both), supposedly to fill the vacuum left by a ‘weak MoH’. The government found its supposedly central role of coordination difficult to perform in the face of the proliferation of donors, among whom there were also difficult interactions and relationships. Donors interfered with all levels of policy development, in an un-coordinated way, as is commonly the case in settings where negotiation capacities over aid are weak [53]. The control of the policy agenda by donors has been well described in other country case studies by Whitfield et al. [[53], chapter 12, ‘aid and power’]. While recipient countries behaved differently from one another, in general donors’ influence in policy processes increased significantly from the structural adjustments period. Some countries were so deeply entangled with donors that in some case it was not possible to even differentiate donors and recipients (e.g. Mali, Tanzania, Ghana, Mozambique and Zambia). Burundi was no different. In many instances, government adopted policy initiated by specific donors. In such a context of complex entanglement between recipients and donors at every level of the policy-making process, the declared commitment to recipients’ ownership is called into question, despite being advocated in the Paris Declaration [15]. As Goldberg and Bryant pointed at, ownership is based on ideological values [30].

### The MoH

The MoH, weakened and fragmented by post-conflict complexities and depleted of resources, did not receive from the donors and from its constituents the necessary trust in order to lead all stakeholders on the path of health sector rehabilitation. The rapidly « moving agenda » at global policy level made it impossible to foster ownership at local level. Ownership, claimed by donors as being so much missing in Burundi, might well be another example of functional tautology as stated by Esser, in order to justify shameless formulation and implementation of donor-driven policy [15]. There was a general agreement in our analysis that the lack of coordination was partly related to the lack of leadership and vision on the part of the MoH, which did not even consider itself a suitable coordinator. Walt et al. noted in other contexts that the MoH cannot be regarded as one sole actor, given the internal divisions following individuals’ affiliation to units or their own interests [8]. In our case study of Burundi, the same applied to the MoH’s ‘central’ and to its ‘vertical programs’ whose dedicated funding undermined any interest in coordination. This suggests that coordination should be understood as a process that needs to take place not only across donors and other country-level recipients, but also within the MoH.

In fragile states, the MoH needs to be strengthened in asserting his authority and legitimacy in order to coordinate [19], but donors, instead, contributed to overwhelm it in Burundi, by unremitting demands. The MoH should be put at the heart of the health system reconstruction process, and not just be a subordinate for externally-decided policies tailored on globally available funding [19].

In Afghanistan, another post-conflict state, NGOs were held accountable to MoH, and a grants and contracts management unit was created, in the MoH, with local people, in order to increase its quality, credibility, in a virtuous circle [31]. Authors showed that with this approach to put the MoH in the driver’s seat, the health system recovered quickly, although there was no mention of the prevailing relationship between donors and MoH [31]. Eventually, the question of lack of trustworthiness of MoH should be investigated further, in light of the post-conflict context, in order to assess its roots. The weak institutional capacity claimed by donors and even by MoH actors probably encompasses this issue of trustworthiness, and there is a need for a deeper understanding of how capacity is defined, built and lost at institutional level.

Last but not least, Lund and Uvin insisted on the necessary shift from clientelism to citizenship in particular in Burundi and change the nature of aid, which is still political in fragile states [17]. Brown et al. showed in their analysis that the World Bank might well play rhetoric at global level (in their pledge to the stalled Health Systems Platform), and this could reflect at country level, particularly in clientelistic approach, but it might also not be the only donor acting as such [54].

## Conclusion

Once again, partners’ organizations put their accountability towards their funders first, instead of putting it towards the MoH and Burundi’s citizens. Adding a sector specific to HIV was the primary mistake, increasing the already complex accountability parallel channels. This resulted in a multiplication of administratively burdensome institutions, chaotic planning and grant applications process, followed by a race for implementation, in which the MoH was sidelined. Ethnic interpersonal issues seemed to have complicated coordination at high level (Ministers). Coordination mechanisms, externally-driven, were not fully grasped, or were sidelined by higher interests.

When tracking the progresses made by Burundi in the IHP+ evaluation report, we see that the compact was eventually signed in December 2012, for the period 2012–2015. The three major contributors to health sector at that time– the BTC, EU and the World Bank - were signatories, as were four UN agencies and three civil society organizations, beside the MoH and the Ministry of Finance. However, as of 2017, it is worrying that USAID was still not signatory, while its contribution to Official Development Aid (ODA) in Burundi increased more than 12-fold between 2007 and 2016. During the same period, other donors such as France, UK or EU, decreased their ODA by 2- to 4- fold [55], the same proportion applying to the health sector [56]. The CCM continues to operate in Burundi as of 2017, mainly managing GFATM-HIV and PEPFAR funding.

The signature of the compact, constituted as a goal for IHP+, might not have succeeded in overcoming the divide between HIV and health sectors, still vivid. Moreover, the compact became a vehicle of the imposition by donors of another global-driven policy, the results-based management in the health sector, mainly illustrated by P4P [57, 58]. Despite numerous high-profile commitments, mostly notably the Paris Declaration, and the establishment of many co-ordination mechanisms these assessments remain fundamentally valid; that aid and aid coordination essentially remained a tool of local and international politics. The whole architecture of aid needs to be transformed, in order to get rid of the possibility of clientelistic relationships running against coordination, and perpetuated by the current nature of aid.

Future research and policy directions in post-conflict countries' aid coordination should focus on two levels: an initial assessment of MoH capacity to coordinate aid towards an effective health system reconstruction, and of the potential for clientelistic relationships, which will be counter-productive to coordination efforts; together with an understanding of the roots of the conflict, which could influence the capacity of MoH to work with people, inside or outside MoH.

## Additional file

Additional file 1: Example of one semi-structured questionnaire for the World Bank Group, at national level (then adapted to each participant). (DOCX 99 kb)

## Abbreviations

BTC: Belgian Technical Cooperation
CCM: Country Coordination Mechanism
DfID: (United Kingdom) Department for International Development
EU: European Union
FCHDP: Framework for consultation of health development partners
GAVI: Global Alliance for Vaccines and Immunization
GFATM: Global Fund for AIDS, Tuberculosis and Malaria
GHIs: Global Health Initiatives
HIV: Human Immunodeficiency Virus
HRH: Human Resources for Health
HSS: Health System Strengthening
IHP+: International Health Partnership Initiative Plus
M&E: Monitoring & Evaluation
MAP: Multisectoral AIDS Program
MDGs: Millennium Development Goals
MoA: Ministry of AIDS
MoH: Ministry of Health
NAC: National AIDS Council
NCAC: National Committee for Aid Coordination
NGO: Non-Governmental Organization
P4P: Pay-For-Performance
PES-NAC: Permanent Executive Secretariat of the National AIDS Council
PR: Principal Recipient
TB: Tuberculosis
UNAIDS: The United Nations Office for AIDS
USAID: US Agency for International Development
USD: United States Dollars
WHO: World Health Organization
