# Supplementary material for: A decade of aid coordination in post-conflict Burundi’s health sector
Source: Global Health. 2019 Mar 29;15:25. doi: 10.1186/s12992-019-0464-z (PMC6440142; doi:10.1186/s12992-019-0464-z)
Supplement: Supplementary file 1 — Example of one semi-structured questionnaire for the World Bank Group, at national level (then adapted to each participant). (DOCX 99 kb) [file 12992_2019_464_MOESM1_ESM.docx]

**Supplementary material**

**Example of one semi-structured questionnaire for the World Bank Group, at national level (then adapted to each participant)**

 What is your position in the WB?

 What are the main responsibilities of your post?

 In general, how does WB work in Burundi?

**A Aid effectiveness**

1) How does WB compare with other funding mechanisms that donors use to provide aid? In what ways? Advantages / disadvantages of each?

2) Does WB work towards harmonisation with other aid mechanisms?

 In what way? What aid instruments do they use?

3) How do partners work together aid effectiveness and coordination?

 What structures/platforms exist to harmonize actions of donors?

*4)* Does the WB work towards aligning themselves with government systems?

*5)* How do you see the government input in coordinating aid?

6) Do you see any tensions in the new aid approach?

**B National policy development**

7) How do you think WB and MAP interact with **national policies, plans and priorities for health**? Please give examples.

8) To what extent is their ‘**ownership**’ in Burundi – by NGOs and other country stakeholders – of the different donors and GHIs operating in this country?

 *What are the reasons for different degrees of country ownership of the GHIs?*

9) What effects have these donors and GHIs had on **intersectoral collaboration?**

**C Financial flow**

10) How do MAP funding flow in Burundi?

11) Are MAP funds channelled through existing mechanisms such as through government ministries or pooling mechanisms such as common fund of the CNLS or SWAp?

12) Is the WB providing **additional funds** in Burundi? Or do you think MAP funds **substituted** for other funds by Government and donors with reduced government commitment to health/HIV/AIDS?

*13)* Do MAP and WBG utilise existing financial management systems or have they established new mechanisms?

14) To what extent do the different partners, including your organisation, get access to funds so as to deliver services?

**D Coordination and planning**

15) What do you see as the major effects of MAP and other donors/GHIs on Government **national planning structures and processes** in Burundi?

 *Do donors and GHIs utilise existing planning and coordination structures such as CNLS, SWAp committees and Interagency Coordination Committees (ICCs), or have they promoted the establishment of new structures (such as CCMs)?*

16) Have donors and GHIs strengthened the functioning of existing structures such as CNLS, helping to focus partner efforts?

 *Explore the balance of positives and negatives – costs and benefits – due to the effects of donors and GHIs on national planning structures and processes*

17) Coming from your national perspective, what do you see as the major effects of these donor initiatives on **district planning structures and processes**?

18) What effects, if any, have donors and GHIs had on **decentralisation policies?**

 *e.g. do they strengthen districts through providing funds to implement district plans?*

 *or are they promoting top-down vertical planning, thereby undermining decentralisation policies*

**E Monitoring and Evaluation**

19) What are reporting mechanisms for MAP? Are you using **existing** reporting and monitoring systems or **separate** reporting requirements? What effects do this have?

*20)* Are there different effects at national and district levels?

**F Human resources**

*21)* What effects have the five different GHIs (Global Fund, World Bank, GAVI) had on Burundi’s Human Resources for Health (HRH) policies?

 *HRH policy formulation for National, Provincial/state, and District levels*

 *Policies for (i) health worker training, (ii) distribution, (iii) remuneration, and (iv) performance management?*

22) What has been the degree of involvement of donors including GHIs on the development of Burundi’s HRH policy and plans?

*23)* To what extent has the Government (MoH) been pro-active in developing HRH policies (and/or to what extent has it been responding to the effects of GHIs*)?*

24) How has MAP affected **health worker availability and performance** in the public health sector?

25) Does MAP actually support **national training capacity**?

 *Training capacity (*number of training institutions and staff)

 *Location of training (rural-urban, tertiary-primary)*

 *Curricula (content and orientation of training: eg selective vs comprehensive; clinical skills vs management/implementation skills)*

26) What **strategies or incentives** does MAP use to attract health workers to work on programmes that you fund? What are levels of motivation of public sector staff compared to non-public staff?

 Salaries and bonuses

 *Training opportunities, continuing education, career development*

 *Recognition and appreciation/ hospital- clinic management*

 *Availability of necessities/ hospital infrastructure*

**Concluding question**

27) How could MAP improve the way they work in Burundi?

 *In what ways? Explore and probe responses*

**What important documents/ reports would you advise me?**

**Who else should I be talking to?**
